# Analyzing changes in parkinsonian speech over time: a diachronic experimental phonetics study

**DOI:** 10.3389/fnagi.2024.1334198

**Published:** 2024-03-12

**Authors:** Massimo Pettorino, Marta Maffia

**Affiliations:** Department of Literary, Linguistics and Comparative Studies, University of Naples L’Orientale, Naples, Italy

**Keywords:** diachronic experimental phonetics, Parkinson’s disease, speech rhythm, longitudinal study, early diagnosis

## Abstract

In this contribution the use of web resources for the longitudinal study of speech rhythm of a ‘well-known’ person diagnosed with Parkinson’s disease, the American actor Alan Alda, is proposed. A corpus of 20 speech samples produced in the period between 1979 and 2021 was collected from the web. A rhythmical analysis was conducted, based on two parameters: the percentage of vocalic portion on the total duration of the utterance (%V) and the VtoV, the mean duration of the interval between two consecutive vowel onset points. The results of this study confirm an early alteration of rhythm in parkinsonian speech, with an abnormal increase of %V, already occurring some years before the clinical diagnosis. The observation of speech rhythm variation can therefore be considered as the basis for the realization of a sustainable and non-invasive procedure in support to early diagnosis of Parkinson’s disease.

## 1 Introduction

This study starts from a very simple consideration: all those who nowadays study speech in all its aspects find themselves in a very favourable position and live an absolutely extraordinary moment. For the first time, in fact, it is possible to hear voices from the past century. The twentieth century is the first to have left sound traces: starting from 1877, the year in which Thomas Alva Edison announced the invention of the phonograph, voices can be recorded, memorized and reproduced on command. History, which until that date had been hopelessly mute and silent, finally becomes sound: to the architectural, pictorial and literary testimonies, sound testimonies are added, such as voices, music, noises. The history of spoken languages becomes a direct object of study: it is now possible to listen, analyse, study the changes that have taken place without having recourse to the mediation of the written text. Such a mediation, in the best of cases, determined in the past an inevitably approximate idea of the real complexity of the speech signal, in which intonation, prosodic variations, accelerations and slowdowns, peaks of intensity, even silent and non-silent pauses, all contribute to conveying a specific message to the listener. Therefore, being able to directly study the voice, without resorting to the written text, makes it possible to move towards a diachronic experimental phonetics. The ‘900 has left us sound archives of all kinds, among which the Internet represents a conspicuous part. How this can help those concerned with phonetics from any point of view is what this study will try to illustrate. As a specific topic for this work, parkinsonian speech will be considered.

## 2 Parkinsonian speech

Parkinson’s disease (PD) is the second most common age-related neurodegenerative disorder (de Lau and Breteler, 2006) and it is diagnosed based on clinical criteria. In recent decades there has been a growing interest in the identification of reliable linguistic and acoustic biomarkers for the diagnosis of PD to be reached as soon as possible, aiding in an early intervention with the most appropriate therapy (Harel et al., 2004; Williamson et al., 2015; Godino-Llorente et al., 2017; Hlavnička et al., 2017).

In addition to various effects on gait, posture, balance and upper limb coordination, the deterioration of dopaminergic neurons in the basal ganglia also provokes an early alteration on speech in PD patients, within a set of characteristics called hypokinetic dysarthria.^1^ The most common speech abnormalities involve hypophonia, changes in voice quality, reduced pitch variability, imprecise articulation, hesitant and disfluent speech, impairment in articulating vowels and consonants (Darley et al., 1969; Forrest et al., 1989; Robertson and Hammerstadt, 1996; Ramig et al., 2008). Furthermore, as far as the rhythm of speech is concerned, many works have found a general dysrhythmia, characterized by an alteration of fluency and of the speech/pause ratio (Liss et al., 2009; Rusz et al., 2015; Lowit et al., 2018). Finally, not always univocal results emerged regarding the acceleration, or slowing down of PD speech. Some authors observed a significative reduction in speech rate in PD patients (Logemann et al., 1978; Ludlow et al., 1987), other reported the opposite variation (Hirose et al., 1982; Ackermann et al., 1995, 1997), other did not find intergroup differences between PD patients and healthy subjects (Duez, 2006a,b; Skodda and Schlegel, 2008).

The observation of such speech alterations in PD can therefore be very useful both in the diagnostic process and in monitoring the progress of the disease and the effectiveness of pharmacological therapy.

## 3 Methodological issues: from synchrony to diachrony

Most of the studies concerning parkinsonian speech and its linguistic and/or acoustic correlates, as well as in the case of other pathologies, are usually conducted with a synchronic approach: the speech produced by a certain number of patients is compared to that produced by a healthy control group, adequately age-and sex-matched. The significance of the results of this kind of studies is tested through statistical analyses. This methodological approach allows the researcher to manage many variables, for example having the same uttered text, verifying the age range of speakers, their linguistic and sociolinguistic characteristics. Therefore, the differences that emerge between the speech produced by one or the other group presuppose changes that must have occurred as a consequence of the unique variable that differentiates the two groups, namely the presence of PD. However, synchronic studies do not allow to evaluate when these changes have occurred. It is indeed not possible to verify what happened before the date of diagnosis, or at least before the emergence of the first symptoms of the disease, as the examined subjects are precisely chosen based on their already ascertained nature of parkinsonian patients.

Still, the need remains for signals that can as soon as possible foresee the onset of the disease, thus allowing for an early diagnosis and the timely starting of therapy.^2^ Is it possible to overcome the temporal limitations of the synchronic approach, going somehow ‘back to the past’? Is there the possibility to follow in real time the onset of those changes found in PD patients in the post-diagnostic period?^3^ To do this, it is necessary to change the research methodology, moving from a synchronic to a diachronic approach. In this regard, various types of resources are today available, first of all certainly the Internet, an accessible, immediate, almost inexhaustible archive of data.

If it is not possible to record a speech corpus of a subject who in the future will be affected by PD, it is actually possible to follow the reverse path, that is to collect and analyse the speech produced by an already ascertained parkinsonian subject and compare it with his own speech produced in the years before diagnosis. It is a path that, to date, it is possible to follow for some subjects, the so-called ‘well-known people’, who have been diagnosed with PD. For them, a large spoken corpus (interviews, public speeches, recited speech) is available on the Internet, as well as on other sound archives. Most of the time it is also possible to obtain the exact year, often even the day, in which a speech sample was produced. Having such a corpus allows the researcher to follow the evolution of the speech of the same subject over time and, thanks to the spectro-acoustic analysis of the signal, to trace the changes that have occurred over the years. No longer, therefore, the problem of finding comparable subjects in terms of age, sex, origin and so on, but only one speaker to be longitudinally examined. In this way it could be possible to see exactly when, compared to the appearance of the first motor symptoms and the consequent diagnosis, the voice begins to undergo verifiable and measurable changes.

Alongside the already highlighted strengths, this type of approach also has some limitations to keep in mind: the spoken text cannot be controlled, and each speech sample is usually pronounced in a different situation, in front of different interlocutors, with different communicative intentions. For this reason, as will be explained later (*cf.* Section 7), it is appropriate to consider utterances that are long enough to avoid occasional variations.

## 4 The rhythm of (PD) speech

As already noted, one of the most evident aspects of PD-related dysarthria concerns an alteration of the rhythm of speech. In previous studies conducted on different languages with a synchronic approach (i.e., comparing PD patients’ speech with that of a healthy control group), the proportion of vocalic intervals in an utterance (%V) was found to be effective in discriminating PD and healthy speakers (Liss et al., 2009; Pettorino et al., 2016, 2017) already in the very early stage of the disease (Maffia et al., 2021). This parameter was firstly introduced by Ramus et al. (1999) for the rhythmic classification of languages: stress-timed languages (e.g., English, German, Polish) are characterized by lower %V values than syllable-timed languages (e.g., French, Italian, Spanish); mora-timed languages (e.g., Japanese, Telugu) have the highest %V values. Although the classification proposed by Ramus et al. has been later discussed and revised, giving rise to a large literature on the subject,^4^ the %V index certainly remains one of the most recurring parameters in the descriptions of speech rhythm.

Another parameter that can be considered for speech rhythm description is the Vowel-to-Vowel index (VtoV), which corresponds to the average interval between two consecutive vowel onsets (Pettorino et al., 2013). In fact, rhythm is linked to the recurrence of audible signal discontinuities. In this regard, a sizeable body of research has demonstrated the existence of prominent moments in the speech signal that are perceptually more salient than others. These “moments of occurrence” correspond to particular points within the syllables, called Perceptual Centers or P-Centers (Morton et al., 1976; Marcus, 1981).^5^ Experimental studies on different languages report that P-Centers tend to coincide with the vowel onsets (Tuller and Fowler, 1980; Barbosa et al., 2005; Pettorino et al., 2015). Consequently, the VtoV interval seems to be the cue that allows listeners to identify the rhythmic pattern of an utterance. It can be considered as the perceptual counterpart of the Articulation Rate (AR), usually measured in syllables/s: the smaller VtoV, the closer the vowels are to each other, and the more accelerated the speech. In other words, VtoV is inversely proportional to AR: VtoV = 1/AR.

On the contrary, the %V index is independent of the AR. It represents a sort of perceptual link between two vowel onset points: the higher the vocalic percentage, the greater the continuity of the speech signal perceived by the listener. Conversely a longer consonantal break determines the perception of a less continuous speech: this is what, in musical terms, goes under the name of *legato* and *staccato* (Maffia et al., 2021). Therefore, to diagram an utterance on the basis of %V and VtoV is a very effective tool to fully represent its rhythmic characteristics. Unlike the traditional method of segmenting speech into linguistic units, such as phones and syllables, too tied to habits and prejudices relating to written language, this new approach takes into account the listeners’ ability to easily perceive the discontinuities along the speech signal.

Following some previous longitudinal studies conducted on limited corpora (Pettorino et al., 2018, 2022), in the present work the examination of a quite large corpus of speech produced by a well-known American actor over 42 years is proposed for the first time. The starting hypothesis is that PD causes a rhythmic alteration in the speech of the observed subject and that this alteration can already be detected several years before the onset of motor symptoms and the subsequent diagnosis.

## 5 Forty-two years of Alan Alda’s speech

Alan Alda is an American actor, director, writer, podcast host, and advocate for science communication. He founded the Alan Alda Center for Communicating Science at Stony Brook University, helping develop innovative programs that enable scientists to communicate more effectively to the public.

On July 31, 2018, while appearing on the CBS Show This Morning, the 82-year-old actor said that he had been diagnosed with PD three-and-a-half years earlier but he had decided to talk about it only in that moment. It is a very special case of early diagnosis, entirely due to the insistence with which Alda asked his doctor to carry out further clinical assessments. These are his words: ‘I had dreamed somebody was attacking me, and in the dream I threw a sack of potatoes at him. In reality, I threw a pillow at my wife’. This encouraged Alda to go to a neurologist for a brain scan. ‘The neurologist examined me and said, “I do not think you need a scan. You do not have any symptoms.” I said, “Well, I’d really like the scan anyway.” And he called me back and said, “Boy, you really got it”’. Months later, he noticed things such as “a little twitch in my thumb”.^6^

Taking into account the peculiar circumstance of a very early diagnosis, it was decided to conduct a longitudinal analysis of Alan Alda’s speech, using the %V/VtoV metrics, in order to verify the presence of rhythmic alterations and to follow their trend over the years, especially in the pre-diagnostic period. To achieve the goal and obtain reliable results from the rhythmic analysis of the utterance, it will be necessary to establish what the minimum duration of the speech samples should be (*cf.* Section 7).

## 6 Materials and methods

A corpus of 2,097 s was analyzed, segmented and labelled using the PRAAT software (Boersma and Weenink, 2023). Spectrographic analysis was carried out on the entire corpus in the frequency range from 0 to 5 KHz, window length 0.05 s. As for the intensity, the dynamic range was adjusted based on the quality of the utterance, generally set at 40 dB.

The selected corpus includes spontaneous, unrecited speech samples. In almost all cases these are television interviews, in which Alan Alda answers the interviewer’s questions or converses with other actors in a round table. In some cases, an audience is also present, in others not.

**Figure 1:**
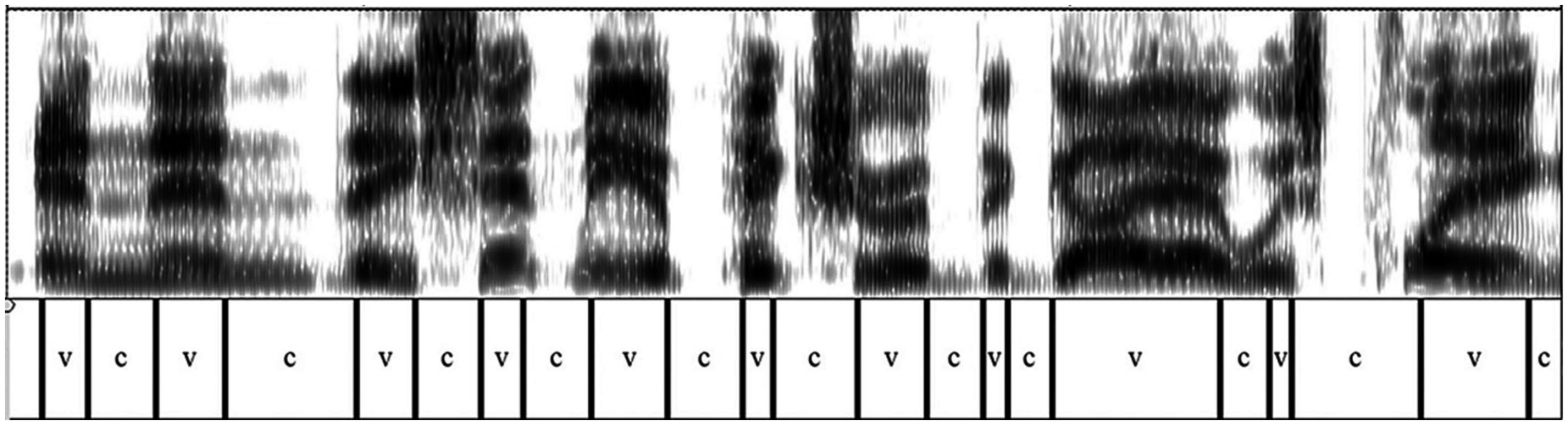
Spectrogram of the sentence “and then they sent me a picture of the guy I was playing” (The Aviator—An Evening with Leonardo DiCaprio and Alan Alda, 2004).

Out of a total of 16,032 intervals, 15,024 were labelled as consonants (C) and vowels (V) for a total duration of 1,561 s. A total time span of 42 years was examined, 36 before diagnosis and 6 after it. The manual annotation of the V and C intervals was conducted by the two authors, who carried out periodic sessions of standardization of the results (in the initial, intermediate and final phases) as well as cross-checks on the performed segmentations. It must be highlighted that this kind of segmentation does not involve particular difficulties, as C and V intervals present very different spectrographic characteristics, which are easily identifiable. Broadly speaking, vocalic sounds are characterized by strong blackening (high airflow due to the free vocal tract) and a clearly distinguishable formant structure (due to the resonances generated in the cavities). On the contrary, consonants show either complete absence of signal (due to the closure of a diaphragm, as in the case of stop consonants) or irregular striations due to the turbulent passage of air through an articulatory constriction, as in the case of fricatives and affricates, or even reduced signal intensity, due to partial closure (as in the laterals) or due to the presence of antiresonances (as in the nasals). However, in a few cases where differences or doubts have arisen in the segmentation, a case-by-case review was carried out and a common procedure established.^7^ The speech samples can be completely downloaded from the Internet (see Appendix). An example of signal segmentation is shown in Figure 1.

The %V and the mean value of the VtoV were then calculated using a PRAAT script. The disfluencies (false starts, nasalizations, vocalizations, etc.) and silent pauses were also noted (by D and X, respectively), although their durations were not considered in the calculation of the %V and VtoV.

Pairwise t-test was used to evaluate the statistical significance of detected changes in %V and VtoV values over years, comparing data in three different time spans, as described in Section 8. The level of significance was set as *p* < 0.05. The analyses were performed with R, version 4.3.1.

## 7 The duration of speech samples

**Figure 2:**
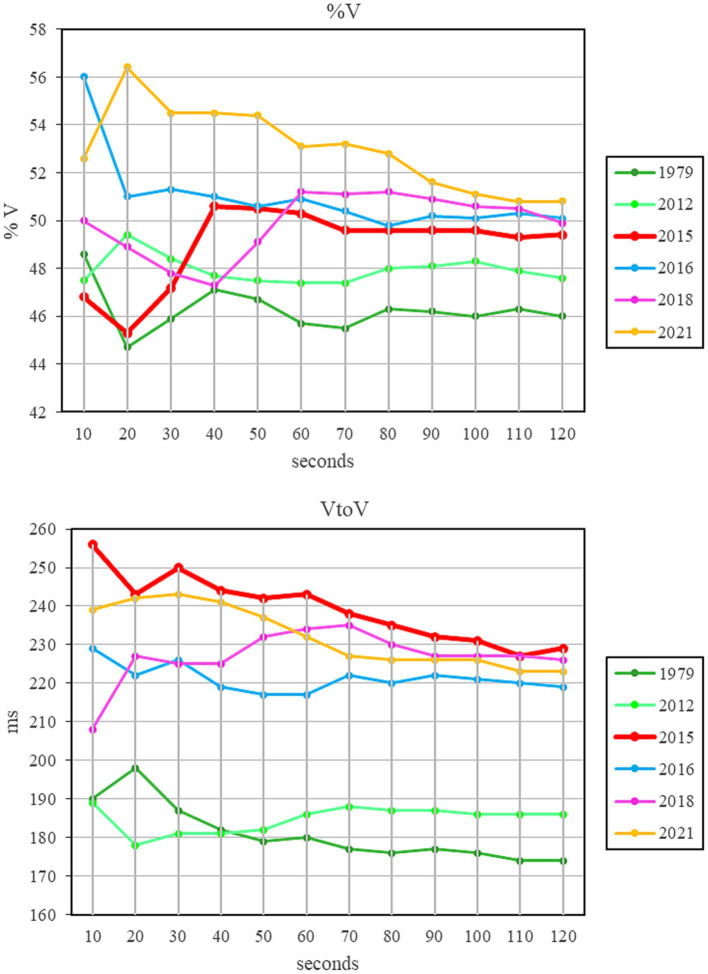
Trend of %V and VtoV relating to sequences of increasing duration in 10 s steps. The red line refers to the year of PD diagnosis.

To avoid including in the corpus too short segments that could give unreliable results due to single anomalous episodes (sequences of several vowels or consonants) and in order to understand how long the analysed sequence should be to obtain reliable and representative data, the following procedure was used: after having segmented the utterance into vocalic and consonantal intervals, VtoV and %V values were obtained relating to sequences longer and longer, starting from 10 s and each time increasing by 10 s. In this way it was possible to verify at which utterance length the values began to stabilize. Figure 2 shows the results of this analysis in 6 time points in the corpus: 1979 and 2021, the two temporal extremes of all data, 2015, the year of PD diagnosis, and three intermediate years.

As can be observed, in the first 40 s there are evident variations in the two indexes (mean Std Dev %V = 1.66; mean Std Dev VtoV = 5.40). The variation tends to decrease in the interval between 50 and 80 s (mean Std Dev %V = 0.59; mean Std Dev VtoV = 2.98). For sequences longer than 90 s the tracing tends to stabilize, with minimal changes in both VtoV and %V (mean Std Dev %V = 0.25; mean Std Dev VtoV = 1.29). Therefore, it was decided to segment sequences longer than 90 s. These sequences include the intervals C and V, as well as silent pauses and disfluencies. The portions of interruptions by the audience (laughter or otherwise) and those in which the interviewer was speaking were not calculated.

## 8 Results

**Figure 3:**
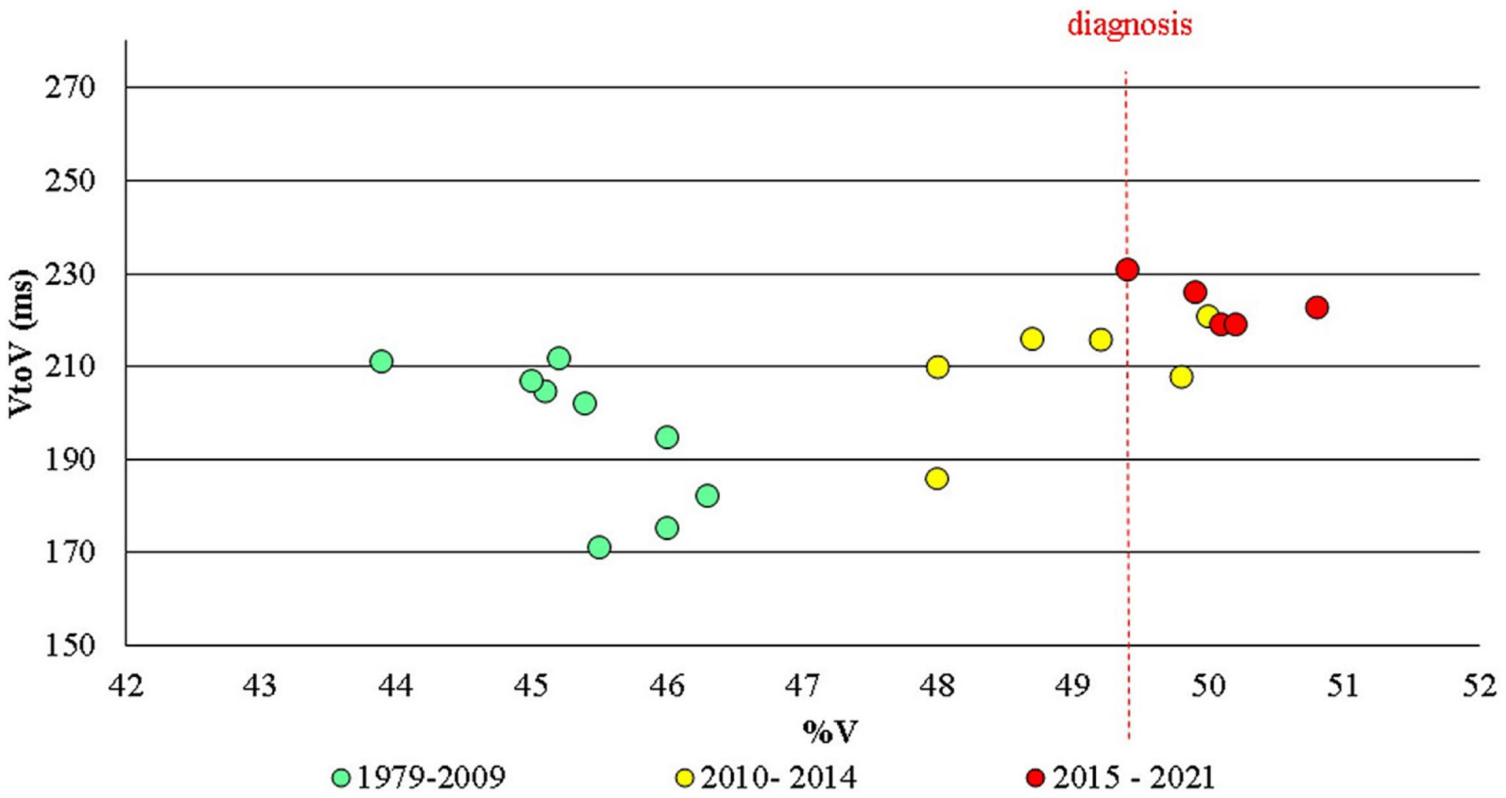
Alan Alda: VtoV and %V for all the speech samples in the corpus in the three time periods examined.

**Figure 4:**
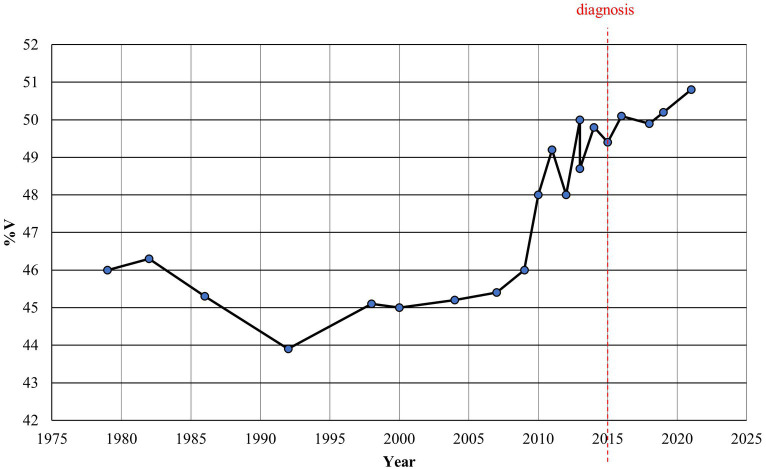
Alan Alda: %V plotted year by year.

**Figure 5:**
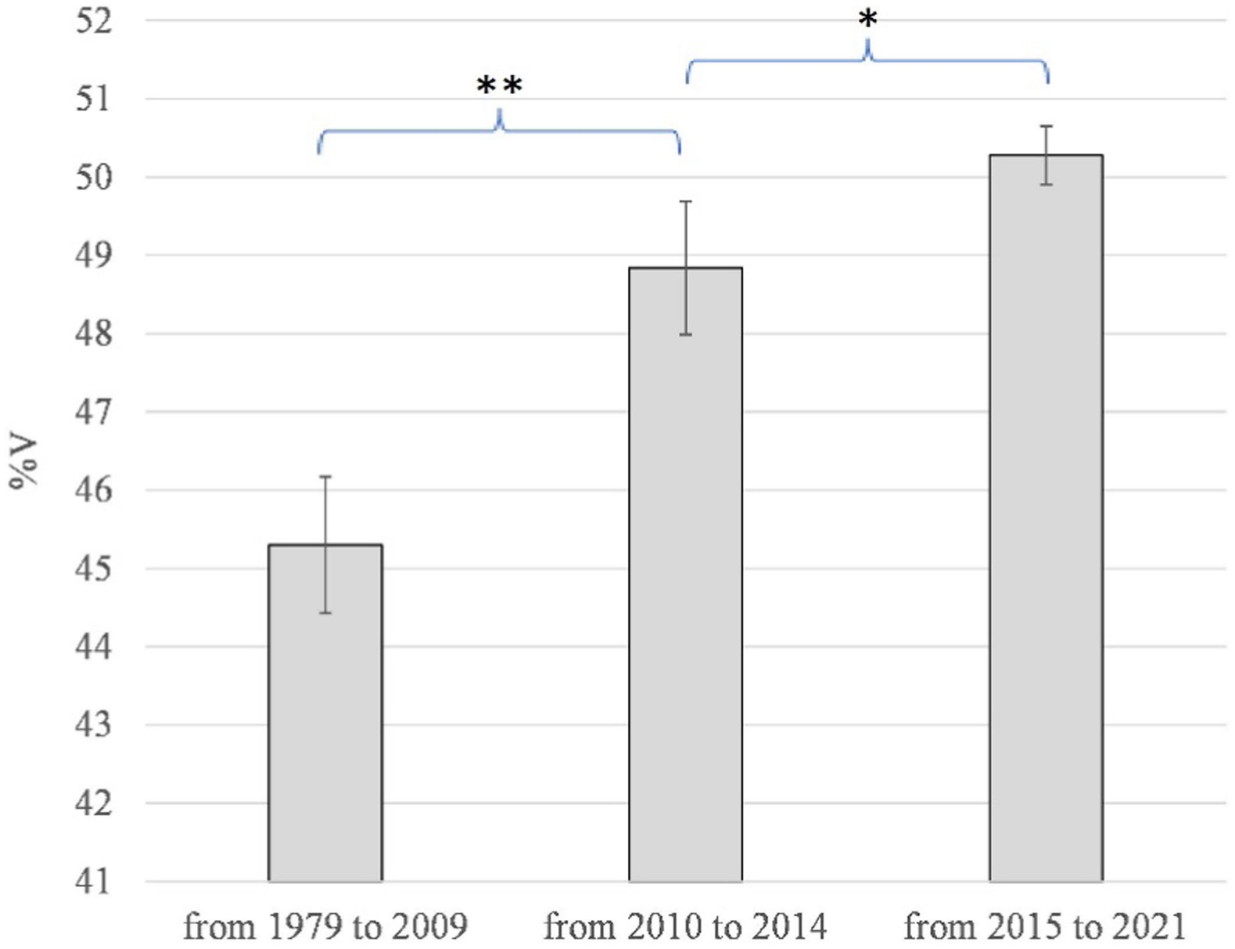
Alan Alda: average values and standard deviations of %V in the three time periods examined. *p ≤ 0.05; **p ≤ 0.01.

The results of the spectro-acoustic analysis are shown in Figures 3–5. The year of diagnosis (2015) was taken as the time reference point; in addition, with a data-driven approach, the pre-diagnostic period was divided into two sub-periods (1979–2009 and 2010–2014), based on the distribution of values as for %V.

As regards the VtoV value, there is a general increase, even if the inhomogeneity of speech styles in the corpus (interviews, public speeches, dialogues) does not allow for well-defined conclusions to be drawn. Nonetheless, it can be noted (see Figure 3) that from 1979 to 2009 VtoV values are between 171 and 212 ms (average value = 196 ms, Std Dev = 16), from 2010 to 2014 they range between 186 and 221 ms (average value = 210 ms, Std Dev =12), from 2015 to 2021 values are between 219 and 231 (average value = 224 ms, Std Dev =5). Statistically, the difference between VtoV values in the period 1979–2009 and those in 2010–2014 is slightly significant (*p* = 0.025); values in 2010–2014 do not result to be different from those in the time span 2015–2021 (*p* > 0.05).

As mentioned above, these VtoV values correspond to an Articulation Rate ranging from 5.1 to 4.7 to 4.4 syllables/s in the three considered time intervals. So, Alan Alda’s speech undergoes a slowdown, of almost half a syllable/s in the 5 years before the diagnosis, that remains quite stable also after 2015.

As for the %V index, Figures 3–5 clearly show that up to 2009 the values are quite constant, with small fluctuations contained in the range 43.9–46.3% (average value = 45.3, Std Dev =0.87). Starting from 2010 until 2014, the values undergo an anomalous increase, going from 48.0% in 2010, to 50.0% in 2013 and 49.8% in 2014 (average value = 48.8, Std Dev =0.85). In the following period, from 2015, the year of diagnosis, to 2021, the increase in %V is confirmed, remaining around 50% (from 49.6 to 50.8; average value = 50.3, Std Dev =0.37). As shown in Figure 5, the difference between the %V values found in the period 1979–2009 and those in 2010–2014 is statistically highly significant (*p* = 0.005); the variation between the %V values in 2010–2014 and those in 2015–2021 is also significant (*p* = 0.012).

If we consider that the first motor symptoms of the disease date back to 2015 with a delay of about 5 years with respect to acoustic data variation, it is evident that the detection of the rhythmic alteration would have supported an even earlier diagnosis of the disease.

## 9 Discussion

The spectro-acoustic analysis of Alan Alda’s speech shows how approximately 5 years before the onset of motor symptoms (as reported by the actor himself) and the consequent diagnosis, the %V parameter undergoes a sudden increase, and then remains more or less constant in the following years, even during the period of pharmaceutical treatment.^8^ These results confirm the findings of the two previous studies (Pettorino et al., 2018, 2022), in which the same methodology was applied on data from two PD speakers diagnosed when aged 30 and 65.^9^ Even in those cases, a sharp increase in %V some years before diagnosis was detected, as long as a subsequent leveling. The sudden jump seems therefore not to be related to advancing age. It can instead be assumed that this rhythmical alteration is due to the typical symptoms of PD with respect to motor activity, such as the difficulty at initiating movements (akinesia), the slowing of the velocity in the execution of movements once initiated (bradykinesia), and the muscular rigidity. Such motor impairments have different effects on the articulation of vowels and consonants. To understand the reasons for this diversity, one must consider the different nature of the two classes of phones.

In the production of a vowel, the channel is open, free. It assumes a determined position, which is very close to the rest position if compared to what happens in the production of consonants. That position is maintained for the entire duration of the phone. Therefore, the vowel is by its nature a ‘static’ phone, which requires very limited motor and neuromuscular activity. On the contrary, the consonant is by its nature a ‘dynamic’ phone, as it requires fast and synchronized movements of the phonatory organs. The articulators, the lips, the tongue in all its parts, the velum, all are involved in producing rapid transitions from diaphragmatic openings to closures or narrowings. Unlike what happens for consonants, in the case of vowels the achievement of a precise and specific articulatory objective is not always required. The proof lies in the fact that the vocalic areas change considerably depending on whether it is a spontaneous speech or a read speech, produced in a formal or informal situation, changes which, however, do not involve any communicative damage.

As a consequence of the unbalanced effort between vowels and consonants, in the dysarthric speech of PD patients vocalic gestures are sustained once they have been started, thus delaying the articulatory passage to the consonantal dynamic phase. Such a prolonging of the static phase accounts for the higher %V in PD speech (Maffia et al., 2021).

It is worth noting that, as reported in Section 4, in a previous study (Maffia et al., 2021) the speech of PD patients and healthy speakers was analyzed using the same metric applied here on Alan Alda’s speech (%V/VtoV). The results of this study, together with those of other works cited in the text (Liss et al., 2009; Pettorino et al., 2016, 2017, 2018, 2022), clarified that the proportion of vowel intervals (%V) is effective in discriminating PD speakers even in the very early stages of the disease. The results of the present study, therefore, can be considered not only typical of a single speaker but, in light of previous data, should be considered of more general reliability.

## 10 Towards a speech test

Looking ahead, a non-invasive speech test capable of detecting an abnormal and sudden increase in %V could be very useful for the clinicians to proceed well in advance with the tests necessary to determine the diagnosis of PD. All these considerations suggest the opportunity to develop a specific software that could automatically segment and label an utterance in consonantal and vocalic intervals, obtaining the %V value. It is worth underlining that such a software should not be configured as a sort of automatic speech recognition nor as a forced alignment task. The speech tool, in fact, would not refer to the uttered text nor to its transcription, but it would exclusively rely on the acoustic characteristics of the signal, performing a segmentation based on the continuous remodeling of the vocal tract due to the dynamics of the articulatory organs and its acoustic correlates.

Such a test will not require any particular, unusual performance from the subject: no vocal interaction with sounds produced by the machine, no requests to produce specific prolonged sounds, nor to pronounce nonsense sentences and words, nor to repeat many and many times a given syllable at a steady pace. What the speaker will be asked to do is simply to record a short passage, in the way that will be most natural to him/her. In short, 90 s for a speech test. It would be an inexpensive, non-invasive and simple-to-administer test, to be also performed in remote conditions. Finally, it must be said that the test would have no claim to constitute a sort of early diagnosis. The subject who, having performed the test, should note a sudden increase in the proportion of vocalic intervals, possibly accompanied by a general slowdown in his own speech production, could simply report this to the doctor and, if necessary, do further medical investigations.^10^

## 11 Conclusion

In this work a new methodological perspective for speech analysis is proposed, moving from a synchronic to a diachronic level and exploiting the sound archive available on the Internet. A speech corpus of the American actor Alan Alda was collected and the acoustic analysis made it possible to identify, 5 years before the date of diagnosis, a sudden abnormal increase in the proportion of vocalic intervals (%V) due to the onset of PD. Furthermore, a progressive slowdown of about half a syllable per second was observed in the corpus, especially starting from the date of diagnosis.

In conclusion, it is worth pointing out that the research that has been here proposed on parkinsonian speech concerns only one of the innumerable fields of research that the immense spoken corpus available on the Internet allows to carry out. The study of ‘voices of the past’ is today an opportunity not to be missed for anyone involved in phonetic research: the experimental analysis of the evolution and changes that have occurred in the last century allows for the first time to study spoken languages without rely exclusively, or predominantly, on written texts.

## Data availability statement

The original contributions presented in the study are included in the article/supplementary material, further inquiries can be directed to the corresponding author.

## Ethics statement

Ethical approval was not required for the studies involving humans because the study was conducted on public data, available on the web. The studies were conducted in accordance with the local legislation and institutional requirements. The participants provided their written informed consent to participate in this study. Written informed consent was obtained from the individual(s) for the publication of any potentially identifiable images or data included in this article.

## Author contributions

MP: Conceptualization, Methodology, Supervision, Writing – original draft. MM: Formal analysis, Funding acquisition, Investigation, Methodology, Software, Writing – review & editing.

## Footnotes

^1^Dysarthria is defined as “a group of related speech disorders that are due to disturbances in muscular control of the speech mechanism resulting from impairment of any of the basic motor processes involved in the execution of speech” (Darley et al., 1975, p. 2).

^2^Many works underline the importance of having a diagnosis as soon as possible (Harel et al., 2004; Williamson et al., 2015; Godino-Llorente et al., 2017; Hlavnička et al., 2017). Among others, a recent study on the use of transcranial sonography (TCS) highlights that “early diagnosis or auxiliary diagnosis of PD in clinical work is crucial for the treatment of PD and the prognosis of patients” (Ma et al., 2022, p. 1).

^3^An attempt to overcome this temporal restriction has been also made by Rusz et al. (2016) in a study involving patients with idiopathic REM sleep behaviour disorder, who are at substantial risk for developing PD.

^4^Among others, see the metrics for the description of the rhythm based on Pairwise Variability Indices (nPVI-V and rPVI-C) proposed by Grabe and Low (2002); rate-normalized interval measures (VarcoV and VarcoC) proposed by Dellwo (2006); Control and Compensation Index (CCI), proposed by Bertinetto and Bertini (2008).

^5^Physical correlates of P-Centres have not been firmly established (Villing et al., 2003) and their exact location is a matter of experimental verification (see Villing, 2010).

^6^CBS This Morning. https://www.youtube.com/watch?v=drGv9fKfjrY.

^7^Given the generally very high level of accord among the two annotators in this simple task, no statistical tests were conducted to assess inter-rater agreement nor reliability.

^8^It should be noted that, as far as treatment is concerned, the present investigation is essentially aimed at the pre-diagnostic period, with the goal of reaching a diagnosis as early as possible and beginning appropriate treatment. Therefore, for the purpose of this research, the observation of the type of medication and the specific analysis of what happens in the speech rhythm after the start of the treatment plan is not relevant as it relates to the post-diagnostic period.

^9^It is intriguing to note that in a longitudinal case-study on a PD subject, a variation in f0 variability was also detected 5 years before the diagnosis (Harel et al., 2004). Further investigation of a larger dataset may confirm the significance of this alteration.

^10^In the complete absence of any motor symptoms, this simple speech test could constitute the first step towards a more accurate clinical assessment. Among the others, a very recent study conducted by Wagner et al. (2023) suggests also the possibility of predicting the onset of PD thanks to the use of data from a 3D eye scan known as “optical coherence tomography” (OCT).

## Appendix

Alan Alda: links from the web to speech samples of the whole corpus.

**Table 1:**

Year	Link
1979	https://www.youtube.com/watch?v=HELG9IIqhBE
1982	https://www.youtube.com/watch?v=RDRSZx-RaHg
1986	https://www.youtube.com/watch?v=6a5lxe7LqHk
1992	https://www.youtube.com/watch?v=gJnPFxaazjw
1998	https://charlierose.com/videos/15359
2000	https://www.youtube.com/watch?v=sJ1mIFbzA6s
2004	https://www.youtube.com/watch?v=VhhXBPiEbts
2007	https://www.youtube.com/watch?v=tJFYytsEynA
2009	https://www.youtube.com/watch?v=MLnAdo-Q5Hc
2010	https://www.youtube.com/watch?v=wHvza4C2iO8
2011	https://www.youtube.com/watch?v=y7A-xL2paOQ
2012	https://www.youtube.com/watch?v=JaV-erc8vOw
2013 (March 5)	https://www.youtube.com/watch?v=659KmoTnehY
2013 (September 7)	https://www.cbsnews.com/news/alan-alda-on-brainy-new-show-and-favorite-mash-episode/
2014	https://aasm.org/video-message-from-alan-alda-encourages-submissions-for-sleep-contest/
2015	https://dianerehm.org/audio/#/shows/2015-07-16/actor-and-science-advocate-alan-alda/110542/@00:00
2016	https://www.goldderby.com/article/2016/alan-alda-horace-and-pete-mash-emmy-the-west-wing-video-13579642/
2018	https://www.youtube.com/watch?v=drGv9fKfjrY
2019	https://www.youtube.com/watch?v=krhRvWCu5IA
2021	https://www.youtube.com/watch?v=abr6CqbNdM4
